# Shear Performance of RC Beams Reinforced with Fe-Based Shape Memory Alloy Stirrups

**DOI:** 10.3390/ma15051703

**Published:** 2022-02-24

**Authors:** Sang-Won Ji, Yeong-Mo Yeon, Ki-Nam Hong

**Affiliations:** Department of Civil Engineering, Chungbuk National University, Cheongju 28644, Korea; tkddnjs0727@chungbuk.ac.kr (S.-W.J.); hong@chungbuk.ac.kr (K.-N.H.)

**Keywords:** Fe-SMA, active confining, shear performance

## Abstract

In this study, the shear performance of a reinforced concrete (RC) beam with Fe-based shape memory alloy (Fe-SMA) stirrups was evaluated experimentally and analytically. Five specimens that had a possibility of shear failure under four-point loading were prepared. The major experimental variables were the spacings (300 and 200 mm) between the Fe-SMA stirrups and whether the stirrups were activated or non-activated. The shear strength of the specimen reinforced with the Fe-SMA stirrups at a spacing of 200 mm was 27.1% higher than that of the specimen reinforced at a spacing of 300 mm. The activation of the Fe-SMA stirrups, which produced active confining pressure, increased the shear strength by up to 7.6% and decreased the number of shear cracks compared to the case of the non-activated specimen. Therefore, the use of Fe-SMA stirrups could significantly improve the usability of concrete members by increasing their shear strength and initial stiffness and by controlling crack formation. Furthermore, finite element method (FEM) analysis was conducted using LS-DYNA, a commercial software program, to predict the shear performance of the RC beam reinforced with the Fe-SMA stirrups. The ultimate load and displacement of each specimen were predicted with errors less than 1.4 and 9.4%, respectively. Furthermore, the FEM predicted the change in failure mode and the stiffness improvement due to the activation of the Fe-SMA stirrups. Therefore, the proposed finite element analysis model can effectively predict the behavior of an RC beam reinforced with Fe-SMA stirrups.

## 1. Introduction

Concrete is most commonly used in the construction of residential facilities and social infrastructure, such as roads, bridges, ports, and dams. Concrete has higher durability and chemical resistance than other construction materials, enabling the construction of structures without restrictions on size and shape [1]. However, concrete is a brittle material, and its tensile and shear strengths are lower than its compressive strength; therefore, it is not possible to construct structural members under bending, tensile, or shear forces, such as beams and slabs, using concrete alone [2]. Reinforced concrete (RC) has been widely used to address these shortcomings of concrete. The steel reinforcement in RC resists the tensile and shear forces caused by external loads. In particular, transverse steel reinforcement is used in RC beams to prevent brittle fracture and ensure the safety and usability of the structure by sufficiently improving the bending strength of the beams [3]. However, the transverse steel reinforcement cannot prevent the occurrence of inclined cracks in the beam, and it begins to resist external forces after the occurrence of cracks [4]. If materials such as salt, moisture, and carbon dioxide penetrate concrete through these cracks, they cause the corrosion of the steel reinforcement, which decreases the load-carrying capacity of the RC member, significantly reduces its durability, and causes concrete spalling [5,6]. Prestressed concrete (PSC) can be used to address the shortcomings of RC. PSC applies compressive force to concrete using tendons before the application of external forces. In particular, inclined tendons increase the shear resistance of concrete as the axial compressive stress decreases the diagonal tension stress caused by shear force. Thus, PSC can control crack formation in concrete effectively and improve the stiffness and usability of the member [7]. However, the use of only prestressing tendons to resist all shear forces acting on a PSC beam is not economical and cannot ensure safety. Therefore, studies have been conducted to improve the shear performance of structures through the external prestressing method [8,9]. In this method, active prestress to a member is introduced by attaching transverse/inclined tendons to the outside of the member. For members reinforced using this method, cracks can be effectively controlled due to the introduction of pre-compressive force, and the stiffness and durability are improved [10]. This method, however, requires the use of anchorage systems in the prestressing process. Such systems complicate the construction process and cause an increase in the construction period, and the tendons used for this method are rapidly corroded due to exposure to the environment [11,12]. Moreover, with this method, it is difficult, or even impossible, to restore prestressing force to the initial value if it is lost over time due to various factors, such as concrete drying shrinkage, creep, and tendon relaxation.

Shape memory alloy (SMA)-based shear reinforcement technology for structures can overcome the shortcomings of the existing methods. A SMA can restore its shape through heating and cooling (activation) despite the occurrence of plastic deformation [13,14]. If pretensioned SMA is activated while its deformation is restrained, it cannot return to its original state, and compressive stress, referred to as recovery stress, is generated in it [15]. When the pretensioned SMA embedded in concrete is activated, recovery stress is generated as the recovery of deformation is inhibited by the bonding force between the SMA and the surrounding concrete. This recovery stress acts as compressive force on the concrete. Based on this principle, prestressed force can be restored through simple reactivation even if it is reduced by various factors. In addition, this technology is easy to apply because no anchorage or jacking system is required; prestressed force is introduced by the bonding force between concrete and SMA.

Among various SMAs, Ni-Ti-based SMA (nitinol) has been most commonly used in industries as it exhibits excellent superelasticity and shape memory effect [16,17]. The main components of nitinol (nickel and titanium), however, are expensive. Moreover, the composition of nitinol must be precisely controlled in the manufacturing process because a slight change in composition leads to an extreme phase change. Therefore, nitinol is costly, and applying it in the construction field is practically impossible [18]. Meanwhile, Fe-based SMA (Fe-SMA) is inexpensive because its main component is iron, and its manufacturing cost is low because it has higher machinability than nitinol. Therefore, various studies have been conducted to apply Fe-SMA in the construction field [19,20,21]. Soroushian et al. [22] experimentally evaluated the shear performance of an RC beam shear-reinforced with Fe-SMA rods. The Fe-SMA rods were placed diagonally on the outside of the RC beam with diagonal tension cracks, and the rods were activated through electrical resistance heating. They reported that the RC beam reinforced with Fe-SMA rods could restore its load-carrying capacity before the occurrence of cracks. Montoya-Coronado et al. [23] experimentally evaluated the shear performance of an RC beam reinforced with Fe-SMA stirrups in the transverse direction. The Fe-SMA stirrups were installed on the outside of the RC beam at 88.9 mm intervals; moreover, the width of the Fe-SMA stirrups, whether the Fe-SMA was activated or non-activated, and whether the beam was reinforced or non-reinforced were considered as experimental variables. They analyzed the results and reported that the shear strength of the RC beam reinforced with the Fe-SMA stirrups was 65–83% higher than that of the non-reinforced specimen. They also reported that the member with activated Fe-SMA showed a smaller number of shear cracks, a larger shear cracking load, and higher stiffness than the member without activation. Ruiz-Pinilla et al. [24] conducted analytical research to predict the shear performance of an RC beam reinforced with Fe-SMA stirrups. They proposed a material model of the Fe-SMA stirrups based on the Ramberg–Osgood model and presented a 2D finite element model (FEM) of the RC member transversely reinforced with Fe-SMA stirrups using the ATENA software. They compared the results predicted using the FEM with the experimental results obtained by Montoya-Coronado et al. [23] to ensure the reliability of the analysis. Based on the analysis, they confirmed that the FEM could predict the crack pattern, maximum shear force, and ductility of the specimen accurately, and the results were consistent with the experimental results.

Most studies that introduce transverse prestressing force in a structure using Fe-SMA, however, are focused on using various types of Fe-SMA on the outside of a member. Studies that introduce transverse prestressing force in a new structure have been scarce. As such, in this study, the shear performance of an RC beam that used Fe-SMA stirrups as shear reinforcement was evaluated experimentally and analytically, and the active prestressing force exerted by the Fe-SMA stirrups was evaluated.

## 2. Experiment

### 2.1. Test Specimens and Variable

**Figure 1:**
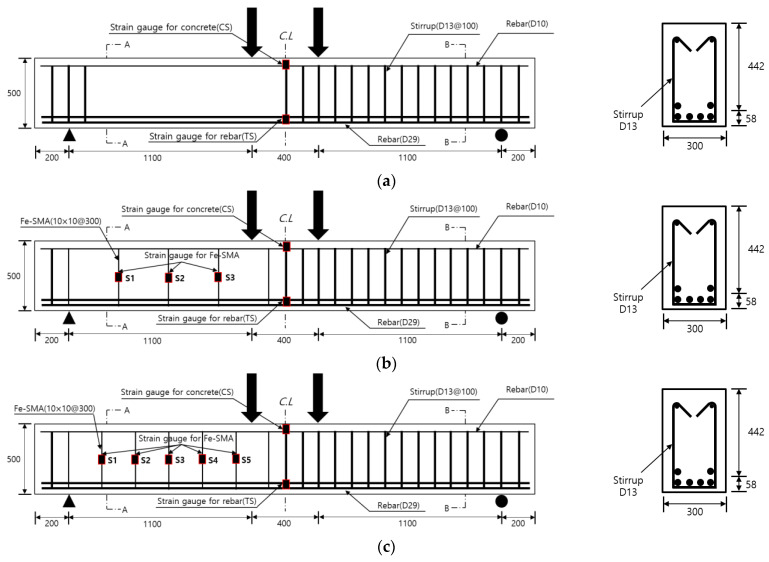
Test specimens. (**a**) CTRL. (**b**) N300 and A300. (**c**) N200 and A200.

A total of five specimens were prepared to evaluate the shear behavior of an RC beam that used Fe-SMA stirrups. As shown in Figure 1, the width, height, and cover depth of the specimens were 300, 500, and 25 mm, respectively. The specimens had a total length of 3000 mm and a clear span of 2600 mm. As shown in Figure 1a–c, each specimen was divided into measurement and non-measurement sections from the center. In the non-measurement section, U-type stirrups made of Φ13 mm SD400 deformed bars were placed at 100 mm intervals to prevent the fracture of the specimen.

The Fe-SMA stirrups used in the experiment were fabricated using Fe-SMA rebars with a square cross-section, which had a side length of 10 mm. The Fe-SMA bars were pre-tensioned to a target pre-strain of 0.04 through horizontal tensioning equipment, and U-type stirrups were prepared from the bars using a rebar bending machine. The Fe-SMA stirrups and rebars were assembled with insulating tape attached to the contact surface between them to prevent power loss during Fe-SMA activation through electrical resistance heating, and copper wires for power supply were installed on the Fe-SMA stirrups. Upon the completion of all preliminary work, ready-mixed concrete was poured into the formwork and subjected to dry curing for 28 days. Table 1 shows the experimental variables considered to evaluate the shear behavior of the RC beam that used Fe-SMA stirrups. The spacing between the Fe-SMA stirrups (non-reinforcement, 200 mm, and 300 mm) and the activation/non-activation of Fe-SMA were considered as experimental variables. In Table 1, the variable name “CTRL” refers to the specimen with no Fe-SMA, and letters “A” and “N” represent the activation and non-activation of Fe-SMA, respectively. The numbers that follow these letters denote the reinforcement spacing of the Fe-SMA stirrups.

### 2.2. Materials

The concrete used to prepare specimens had a design strength of 40 MPa. For this concrete, the maximum size of coarse aggregate was 25 mm, the fine aggregate ratio was 47%, and the water–binder ratio was 30.7%. To measure the compressive strength of this concrete, five cylindrical samples with a size of Φ100 mm × 200 mm were fabricated from this concrete. These samples were demolded and cured under the same conditions as the specimens. On the day of the experiment, the compressive strength of the concrete measured in accordance with the ASTM standards [25] was 46.2 MPa.

The rebars used as tensile rebars, compressive rebars, and shear stirrups were SD400 deformed bars. Their nominal diameters were 28.6, 9.53, and 12.7 mm, respectively. Table 2 shows the material properties of these rebars provided by the vendor.

**Figure 2:**
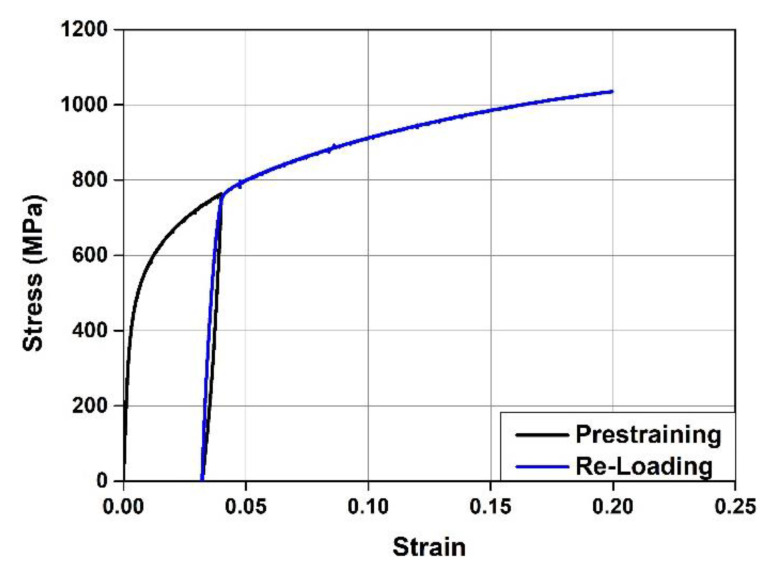
Stress–strain curves of Fe-SMA.

The chemical composition of the Fe-SMA used in this study was Fe-17Mn-5Si-5Cr-0.3C-4Ni-1Ti. To measure the mechanical properties of this Fe-SMA, test pieces with a width of 10 mm, thickness of 2.5 mm, and length of 200 mm were prepared. As mentioned previously, the Fe-SMA stirrups were fabricated using Fe-SMA bars with a pre-strain of 0.04. As such, the Fe-SMA test pieces were tensioned with a displacement control of 0.25 mm/min using a 100 kN universal testing machine (UTM) (Instron, Norwood, MA, USA) until a pre-strain of 0.04 was reached, and then it was unloaded until the load became zero. A direct tensile test was conducted to evaluate the mechanical properties of the pre-strained test pieces, with a displacement control of 0.5 mm/min. During the test, the strain of the test piece was measured using a strain gauge, and the measured data were collected every second through a data acquisition system (DAQ) (Tokyo Sokki Kenkyujo, Tokyo, Japan). Figure 2 shows the stress–strain relationship of Fe-SMA obtained through the direct tensile test. When the test piece tensioned to a pre-strain of 0.04 was unloaded, the residual strain generated in it was 0.032. When it was loaded again, the stress–strain relationship returned to the previous pre-strain point. Finally, the test piece was ruptured at approximately 1035 MPa with an elongation of approximately 20%. As shown in Figure 2, Fe-SMA does not show a typical yield point unlike general steel. Therefore, a 0.2% offset method was used to estimate the yield point of pre-strained Fe-SMA. The yield strength of the pre-strained Fe-SMA calculated likewise was about 720 MPa, and the strain was about 0.039.

**Figure 3:**
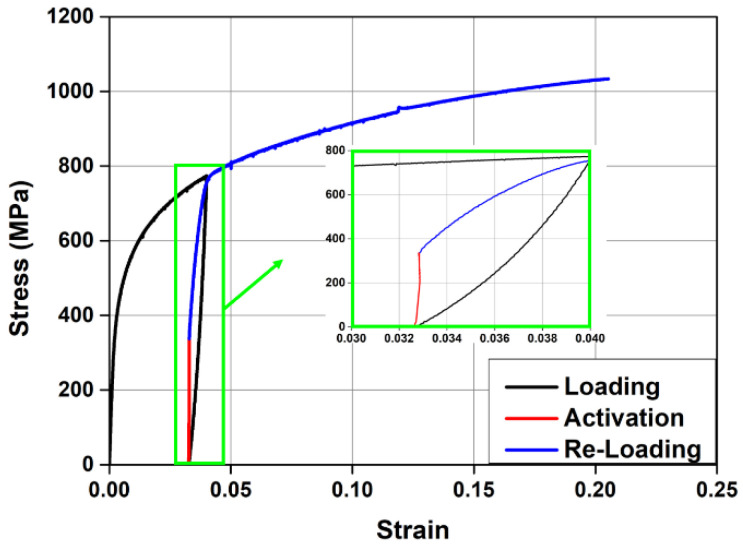
Stress–strain curves of Fe-SMA.

**Figure 4:**
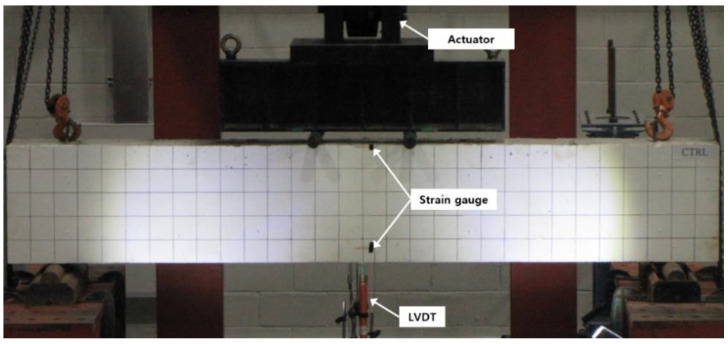
Test setup.

The test to evaluate the recovery stress was conducted in the following sequence:

(1) The test piece placed in the 100 kN UTM was tensioned up to a pre-strain of 0.04 and then unloaded in the same manner as mentioned above (black line in Figure 3);
(2) The test piece with the residual strain generated after unloading was subjected to a pre-stress of approximately 50 MPa to prevent the buckling caused by initial thermal expansion, and then its displacement was restrained;
(3) The test piece with restrained displacement was heated to 160 °C through electrical resistance heating by supplying a current of 2 A/mm^2^ and then cooled to room temperature (red line in Figure 3);
(4) The test piece cooled to room temperature was tensioned with a displacement control of 0.5 mm/min until it was ruptured (blue line in Figure 3). During the activation of Fe-SMA, the temperature of the test piece was measured by an infrared heat sensor, and the measured data were collected every second by the DAQ.

As shown in Figure 3, when the temperature of the test piece heated to 160 °C reached room temperature, the recovery stress generated in it was approximately 335 MPa. When it was loaded again, the stress–strain relationship returned to the previous pre-strain point. After this point, the stress–strain relationship of the test piece was similar to that of the non-activated test piece.

### 2.3. Test Setup

The Fe-SMA stirrups embedded in the specimens were activated through electrical resistance heating by supplying a current of 5 A/mm^2^. Since contact-type thermocouples generally measure the test piece temperature through a resistance change, they cannot accurately measure the temperature of an object in which the current flows. Moreover, as the Fe-SMA stirrups are embedded in concrete, it is also impossible to measure the temperature with non-contact heat sensors. Therefore, a preliminary experiment was performed to measure the time required for the Fe-SMA stirrup surface to reach 160 °C when a current of 5 A/mm^2^ was supplied. It was confirmed that the stirrup surface reached 160 °C when a current of 5 A/mm^2^ was supplied for 26 s. Therefore, the Fe-SMA stirrups embedded in the specimens were heated by supplying a current of 5 A/mm^2^ for 26 s. After the heated Fe-SMA was sufficiently cooled, a four-point loading test was conducted to evaluate the shear performance of the concrete beam using a 2000 kN actuator. As shown in Figure 1, the distance between the loading points on each specimen was 400 mm, and each loading point was 200 mm away from the center of the specimen in both directions. In the test, loading was applied at a rate of 1 mm/min through displacement control. The deflection of each specimen due to loading was measured using two linear variable differential transformers (LVDTs) with a capacity of 200 mm, which were installed at the lower center of the beam. The strains of the concrete, tensile rebars, compressive rebars, and Fe-SMA stirrups were measured using strain gauges. However, the stirrup strains of the specimens in which the Fe-SMA stirrups were activated were not measured due to damage to the strain gauges during electrical resistance heating. In addition, initial crack formation and crack propagation that occurred in the specimens during loading were visually observed and recorded on the surface of the specimens. Figure 4 shows the setup of the four-point bending test.

## 3. Experimental Result and Discussion

### 3.1. Failure Mode

**Figure 5:**
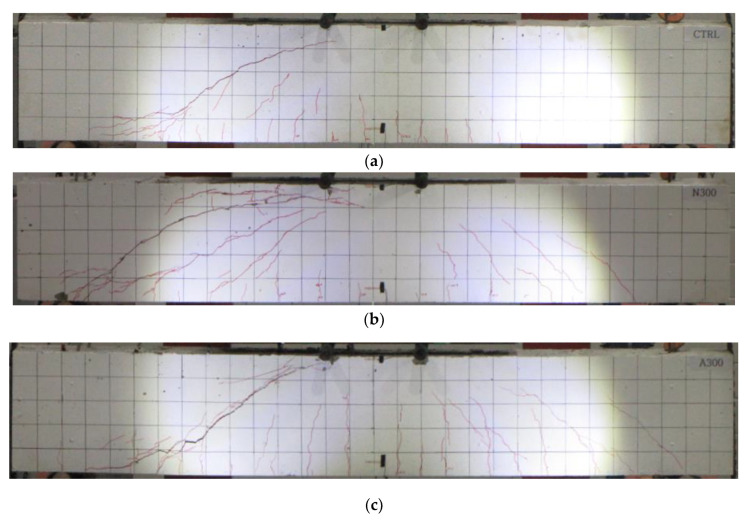

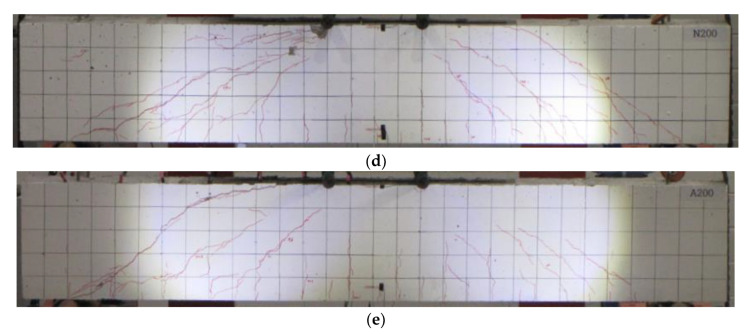
Failure mode of specimens. (**a**) CTRL. (**b**) N300. (**c**) A300. (**d**) N200. (**e**) A200.

Figure 5 shows the failure modes of the specimens upon the completion of the test. As shown in Figure 5a, CTRL with no Fe-SMA stirrup exhibited a typical shear failure pattern due to diagonal tension cracks in the test section after the occurrence of flexural cracks in the center of the beam. The failure modes of all the specimens were similar to that of CTRL. N300, a specimen with non-activated Fe-SMA stirrups, however, showed more diagonal tension cracks than A300, a specimen with activated Fe-SMA stirrups. The activation of the Fe-SMA stirrups decreased the number of diagonal tension cracks due to the generated recovery stress in A200 and N200, for which the reinforcement spacing of the Fe-SMA stirrups was 200 mm. It appears that the number of diagonal tension cracks decreased because the recovery stress generated by the Fe-SMA stirrup activation acted as active confining pressure on the specimens.

### 3.2. Load–Deflection Relationship

**Figure 6:**
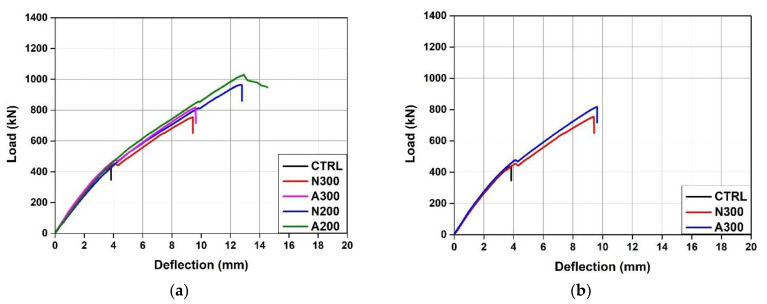

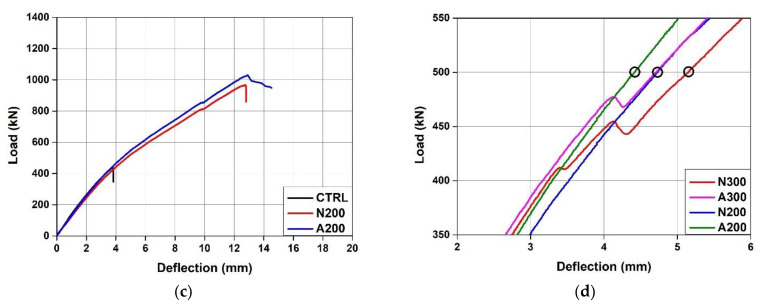
Comparison of load-deflection curves. (**a**) All specimens. (**b**) N300 and A300. (**c**) N200 and A200. (**d**) At 500 kN.

Table 3 shows the summary of the test results, and Figure 6 shows the load-displacement relationship of each specimen. The initial cracking load of the CTRL specimen showed a small difference of 3.6% compared to those of the specimens reinforced with Fe-SMA stirrups. This indicates that transverse reinforcement through the Fe-SMA stirrups had no significant impact on the initial cracking loads of the specimens. The ultimate loads of N300, A300, N200, and A200 were 72.51, 87, 120.86 and 135.95% higher than that of the CTRL specimen, respectively. In particular, as shown in Figure 6b,c, the ultimate load of A300, a specimen with activated Fe-SMA stirrups, was approximately 8.4% higher than that of N300, and the ultimate load of A200 was 6.8% higher than that of N200. This appears to be because the recovery stress generated in the Fe-SMA stirrups due to the Fe-SMA activation acted as active confining pressure on the specimens and increased their shear strengths.

Moreover, as shown in Figure 6b, the load of the N300 specimen was reduced by about 11.56 kN with the occurrence of diagonal tension cracks at 455 kN, which exceeded the CTRL ultimate load of 437 kN. The A300 specimen also showed a decrease in load of about 9.28 kN with the occurrence of a diagonal tension crack at about 477 kN. However, this was not observed in N200 and A200, for which the reinforcement spacing of the Fe-SMA stirrups was 200 mm. This is considered to be because the shear force was not sufficiently transmitted to the Fe-SMA stirrup as sufficient reinforcement was not present in A300 and N300, for which the reinforcement spacing of the Fe-SMA stirrups was 200 mm.

Figure 6d compares the displacements of the specimens reinforced with the Fe-SMA stirrups under a load of 500 kN. When a load of 500 kN was applied, N300 showed a displacement of 5.14 mm. Under the same load, the displacements of A300, N200, and A200 were 8.4%, 8.0%, and 14.2% lower than that of N300, respectively. This confirmed that it is possible to improve the stiffness of a specimen by increasing the amount of transverse reinforcement or introducing transverse prestressing force through Fe-SMA activation. Therefore, the RC beam constructed with Fe-SMA stirrups is expected to exhibit an improvement in shear strength and initial usability.

### 3.3. Load–Strain Relationship

**Figure 7:**
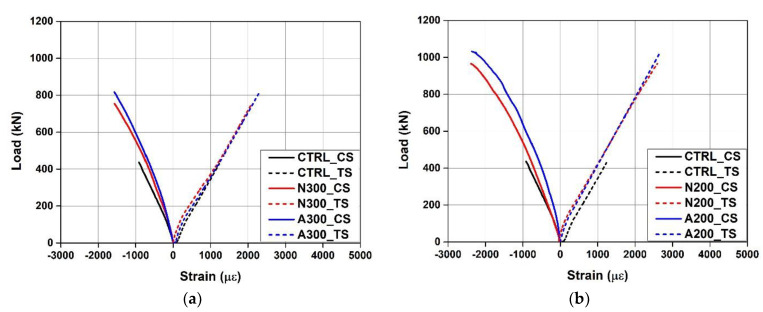
Comparison of load–strain curves. (**a**) N300 and A300. (**b**) N200 and A200.

**Figure 8:**
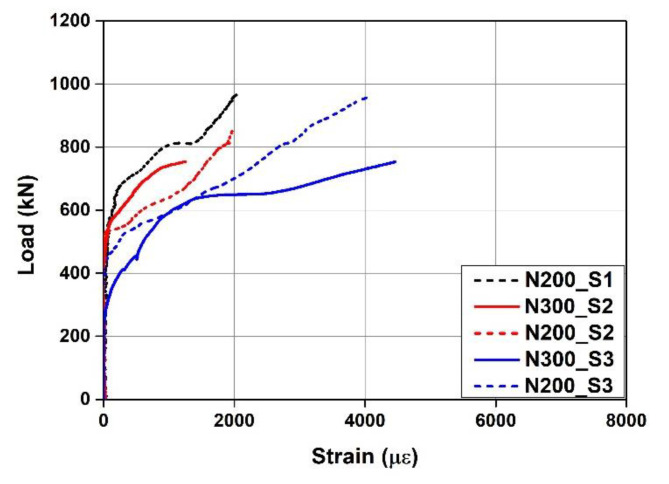
Comparison of load–Fe-SMA stirrup strain curves for N200 and N300.

Figure 7 shows the load–strain curves of the concrete and rebars measured during the test. “CS” and “TS” in the legend represent the strain of the concrete in the compressed part and the strain of the tensile rebars, respectively. As can be seen from the figure, the strain of the tensile rebars did not exceed the yield strain of 2400 με for all the specimens. The strain of the concrete also did not yield. Therefore, it was confirmed that the tensile rebars and concrete of the specimens did not yield, and the specimens were ruptured by concrete shear failure. Figure 8 shows the load-Fe-SMA stirrup strain curves of N300 and N200 with the non-activated Fe-SMA stirrup. In this instance, the strain of N300 at position S1 and the strains of N200 at positions S4 and S5 were not measured due to damage to the strain gauges attached to the specimens. At the time point when N300 was subjected to shear failure, the Fe-SMA stirrup strains at S2 and S3 were found to be 1252 and 4457 με, respectively. At the time point when N200 was subjected to shear failure, the Fe-SMA stirrup strains at S1 and S3 were found to be 2027 and 4023 με, respectively. At S2 of N200, the Fe-SMA stirrup strain was measured only until 851 kN before the ultimate load was reached, and the strain was found to be 2005 με.

## 4. Finite Element Simulation

### 4.1. Finite Element Model

**Figure 9:**
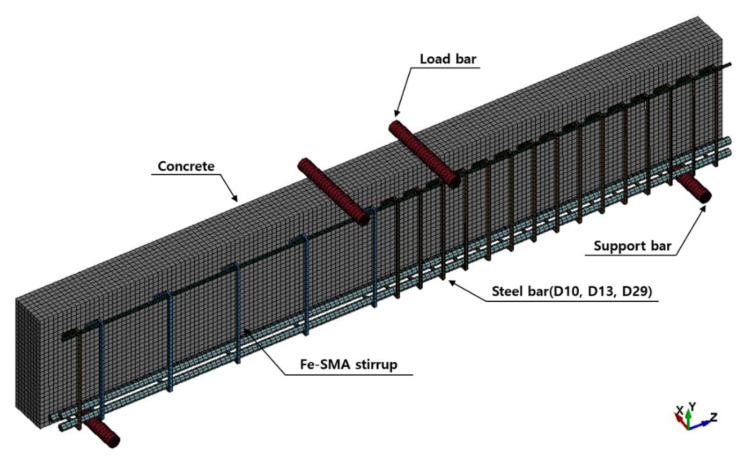
Modeling geometry.

Figure 9 shows a finite element (FE) analysis model to predict the shear behavior of the RC beam reinforced with the Fe-SMA stirrups. FE analysis was conducted using LS-DYNA [26], a commercial FE analysis software program. Concrete was modeled with an eight-node integration solid element with a size of 20 mm, while the rebars and Fe-SMA stirrups were modeled with a two-node Hughes–Liu beam element with a size of 25 mm. The supports and loading points were modeled with rigid elements to prevent the concentration of loads. The rebars, stirrups, Fe-SMA bars, and concrete were assumed to be completely attached. To implement this, the Lagrange in solid command was used. In addition, the automatic surface to surface command was used to prevent the infiltration of the support and load bars into the concrete solid element.

The analysis was conducted using displacement control based on the Newton–Raphson method, which calculates the internal force by applying vertical displacement to the load bars. During the displacement control analysis, the increment in displacement was set to 0.001 mm per step. The analysis was conducted until the displacement of the load bars reached 25 mm.

### 4.2. Material Model

Table 4 shows the material model input values of the concrete, rebars, Fe-SMA, load bars, and support bars used in this study. The models in LS-DYNA were used as the material models for FE analysis. MAT_SCHWER_MURRAY_CAP was used as the material model of concrete. This model can simulate the softening and hardening behavior of concrete, as well as its compressive, and shear behavior. It determines input values according to the compressive strength of concrete, aggregate size, and element size [27]. The constitutive equation of this model is given by Equation (1). $F_{f}$ is the maximum yield surface, which is a function of the stress-invariant $I_{1}$.

(1) $$F_{f}=\alpha -\gamma \times e^{-\beta I_{1}}+\theta I_{1}$$

where α, β, γ, and θ are parameters for shear failure according to the compressive strength of concrete. They were calculated using the method proposed by Jiang et al. [27].

The piecewise linear plasticity model was used as the material model of rebars. This model can perform the modeling of elastoplastic behavior according to the arbitrary stress–strain relationship and simulate the behavior according to the strain rate. In this study, rebars were modeled using the material properties shown in Table 2.

**Figure 10:**
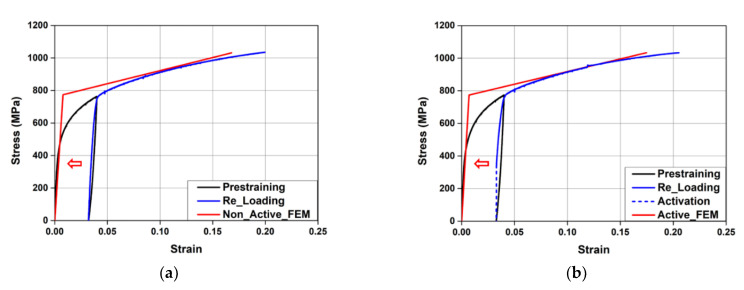
Stress–strain curves of Fe-SMA used in the FE model. (**a**) Non-activation. (**b**) Activation.

**Figure 11:**
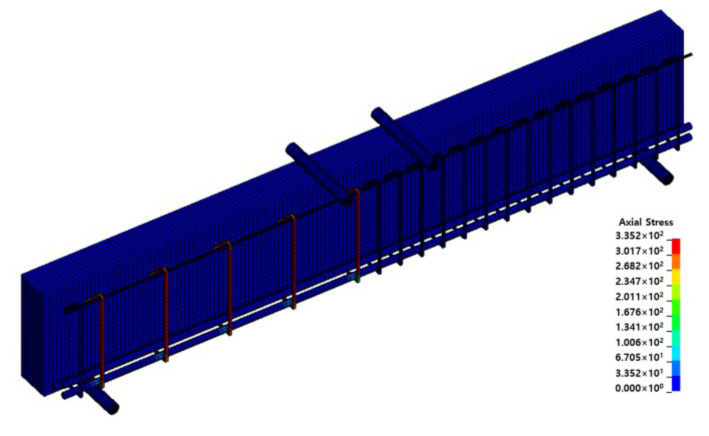
Recovery stress according to thermal load.

LS-DYNA does not support prestressed material models. Therefore, some researchers proposed methods to implement prestress using the MAT_ELASTICI_PLASTIC_THERMAL model, which can consider the temperature range and thermal expansion [28]. As such, MAT_ELASTICI_PLASTIC_THERMAL was also used as a Fe-SMA material model in this study to simulate the recovery characteristics due to Fe-SMA activation. This model can express the shape recovery of Fe-SMA due to activation through the thermal expansion and contraction caused by the temperature load. In this instance, the stress–strain relationship of Fe-SMA according to activation or non-activation was applied by offsetting the residual strain that was generated after prestraining as the initial strain, as shown in Figure 10a,b. The temperature-induced strain of this model is given by Equation (2) [29].

(2) $$\varepsilon_{T}=\Delta T\alpha$$

where ΔT is the temperature change (°C) and α is the thermal expansion coefficient. A thermal expansion coefficient of −21.4 × 10^−6^ was applied to simulate the recovery strain according to the Fe-SMA temperature increase, and ΔT was set as 140 °C, obtained by subtracting room temperature (20 °C) from the heating temperature (160 °C). As shown in Figure 11, when 140 °C was applied, the compressive stress acting on the Fe-SMA stirrups was the same as the recovery stress (335 MPa).

### 4.3. Comparison of Experimental and Analysis Results

#### 4.3.1. Failure Mode

**Figure 12:**
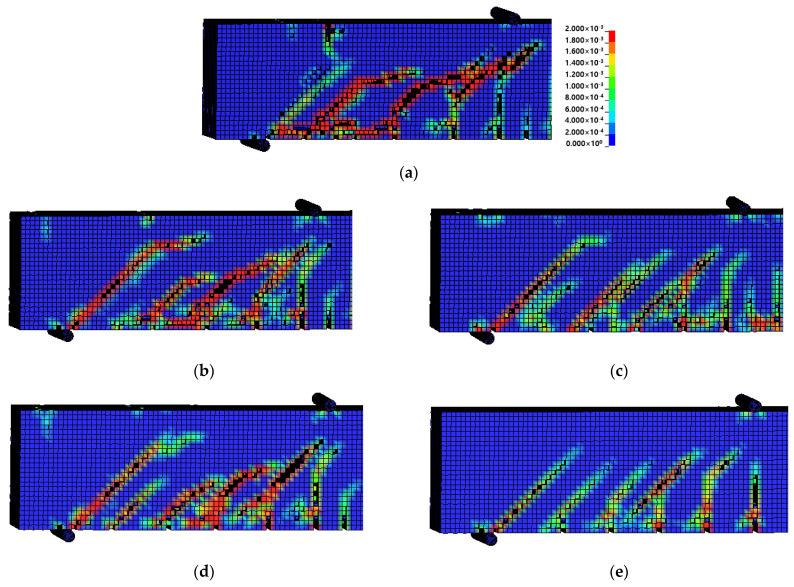
Failure mode. (**a**) CTRL. (**b**) N300. (**c**) A300. (**d**) N200. (**e**) A200.

Figure 12 shows the failure modes of the specimens in the test section predicted through FE analysis. These failure modes were confirmed through the average strain of concrete. If the strain of a concrete element exceeded the limit of the concrete plastic strain, the element was deleted. With regard to the failure modes of all the specimens, shear failure due to the diagonal tension cracks caused by the increasing load after the occurrence of initial cracks at the lower center of the specimen was predicted in the same manner as in the experiment. As shown in Figure 12a, the failure mode of CTRL predicted through FE analysis was the shear failure caused by a rapid brittle fracture in the test section, which was similar to the test results. The failure modes of the specimens reinforced with the Fe-SMA stirrups are shown in Figure 12b–e. The A300 and A200 specimens, which had activated Fe-SMA, exhibited lower concrete strains and reduced crack patterns than N300 and N200, which had non-activated Fe-SMA. Therefore, it was judged that the proposed

FE analysis model could predict the failure mode of the RC beam reinforced with Fe-SMA stirrups fairly accurately.

#### 4.3.2. Load–Deflection Relationship

**Figure 13:**
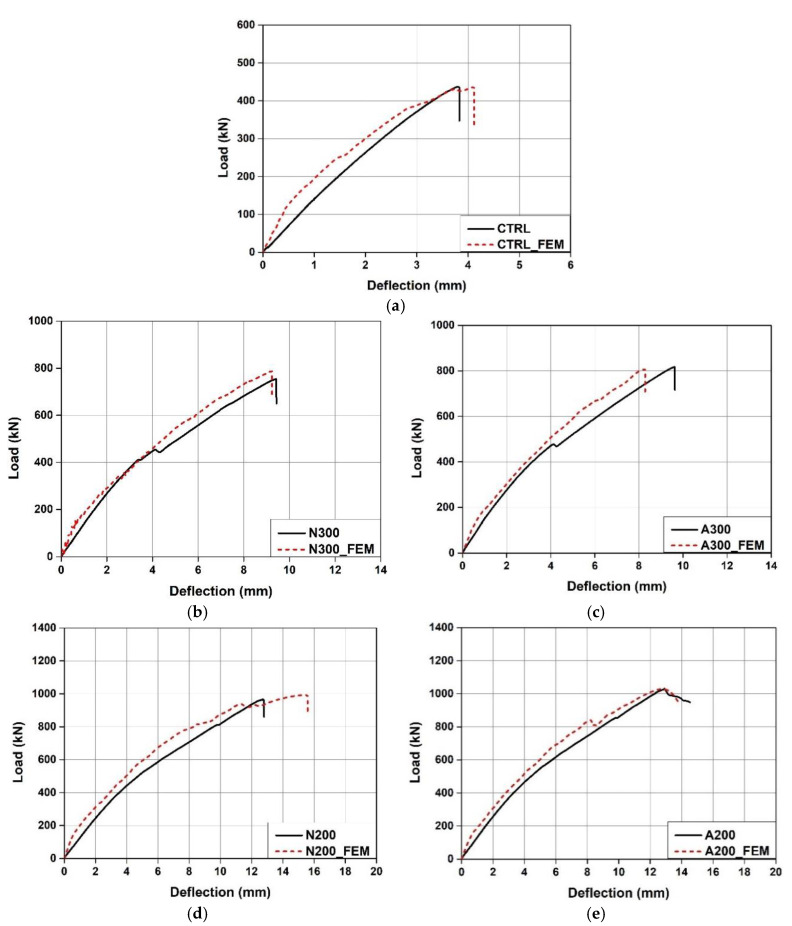
Comparison of load–deflection curves between experiment and FE model. (**a**) CTRL. (**b**) N300. (**c**) A300. (**d**) N200. (**e**) A200.

Table 5 summarizes the FE analysis results of the concrete beam fabricated using the Fe-SMA stirrups, and Figure 13 compares the load–displacement relationship of each specimen measured by the experiment with that predicted by FE analysis. As shown in Figure 13, the load–displacement relationships of the specimens predicted through FE analysis were similar to the experimental results. The FE analysis results predicted that CTRL would be ruptured by concrete shear failure at 436.01 kN. The difference between this failure load and of the failure load determined in the test (437.08 kN) was only 0.2%. This indicates that the concrete material model used for FE analysis in this study can predict the shear failure of concrete accurately. As shown in Figure 13b,c, the ultimate loads of N300 and A300 were predicted to be 788.63 and 808.13 kN, respectively. These values differed from the experimental results only by 4.6% and 1.1%, respectively. In addition, the ultimate loads of N200 and A200 as predicted through FE analysis differed from the experimental results only by 2.8% and 0.3%, respectively. The ultimate load of A300 predicted through the analysis was 992.49 kN, which was approximately 2.5% higher than that of N300. The ultimate load of A200 was predicted to be approximately 3.6% higher than that of N200. Moreover, when a load of 500 kN was applied, the displacements of N300 and A300 were 4.49 and 3.94 mm, respectively, indicating that the displacement decreased by approximately 12% at 500 kN due to the activation of the Fe-SMA stirrups. When N200 and A200 were loaded, they exhibited displacements of 3.97 and 3.86 mm, respectively. Thus, the activation of the Fe-SMA stirrups decreased the displacement by approximately 3% at 500 kN. Therefore, it is judged that the proposed FE analysis model can accurately predict an increase in the shear strength and stiffness of the beam due to Fe-SMA stirrup reinforcement and activation.

#### 4.3.3. Load–Fe-SMA Stirrup Strain Relationship

**Figure 14:**
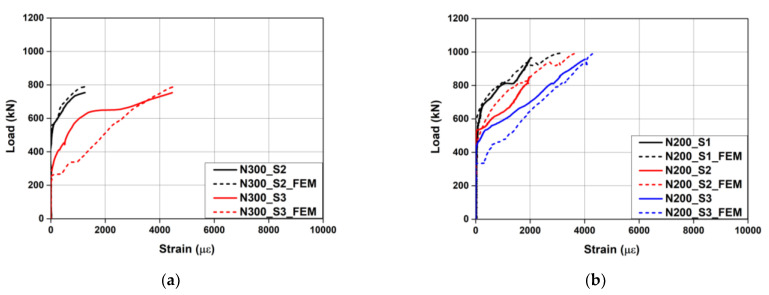
Comparison of load–Fe-SMA stirrup strain curves obtained from the experiment and FE model. (**a**) N300. (**b**) N200.

Figure 14 compares the load–Fe-SMA stirrup strain relationships measured through the experiment with those predicted through FE analysis. In the analysis, when N300 reached the ultimate load, the strains at S2 and S3 were predicted to be 1233 and 4574 με, respectively. These values differed from the experimental results only by 1.5% and 2.6%, respectively. In the case of N200, when the ultimate load was reached, the strains at S1 and S3 were predicted to be 2627 and 4110 με, respectively, which differed from the experimental results by 22.8% and 2.1%, respectively. The average difference in Fe-SMA stirrup strain between the experimental and analysis results at the ultimate load was 7.3%, indicating that the analysis could predict the Fe-SMA stirrup strain accurately.

**Figure 15:**
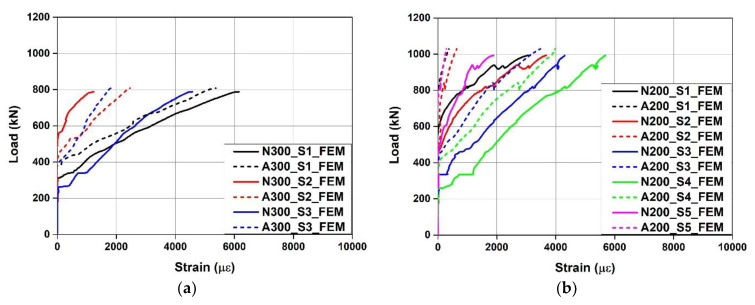
Load–Fe-SMA stirrup strain relationships predicted by FE model. (**a**) N300 and A300. (**b**) N200 and A200.

Figure 15 shows the load–Fe-SMA stirrup strain relationship predicted through FE analysis. The loads of A300 and A200 at the time of strain increase were predicted to be 382 and 507 kN, respectively, which were 45.5% and 20.2% higher than those of N300 and N200, respectively. In addition, as the Fe-SMA stirrups were activated, the Fe-SMA stirrup strain under the ultimate load generally decreased, and major deformation occurred at positions A300_S1, A200_S1, and S4 compared to the constant strain distribution observed from the specimens with non-activated Fe-SMA stirrups. These results confirmed that the activation of Fe-SMA stirrups can delay the occurrence of shear cracks. This appears to be because the active confining pressure generated by the recovery stress was exerted on the inside of the concrete beam.

### 4.4. Verification of the Analysis Model

The proposed FE analysis model was verified through a comparison with the results of a study conducted by Czaderski et al. [12]. The specimen used in their study was a T-shaped beam with a height of 750 mm, flange width of 650 mm, flange height of 150 mm, and web width of 160 mm. The T-shaped beam was externally reinforced with Fe-SMA strips, and Fe-SMA activation/non-activation and the Fe-SMA strip width were considered as test variables. The material model presented in Section 4.2 was used to verify the proposed FE analysis model, and the numerical values in references [12] were used for the properties of each material.

**Figure 16:**
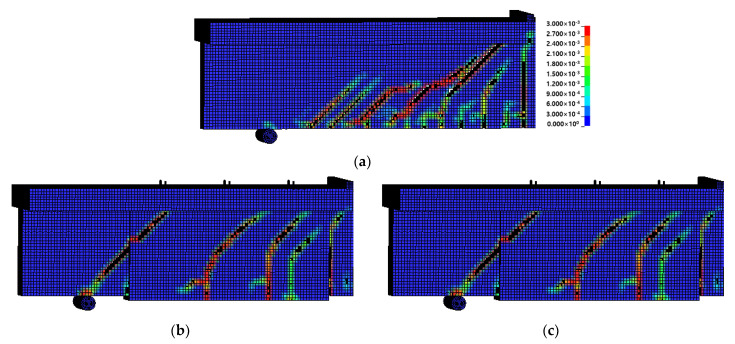
Failure mode predicted by FE model. (**a**) Beam 1. (**b**) Beam 2. (**c**) Beam 3.

Figure 16 shows the failure modes of the specimens as predicted by FE analysis. The failure mode of the concrete beam reinforced with Fe-SMA strips predicted by FE analysis was found to be shear failure, similar to that reported by Czaderski et al. [12].

For Beam 1, a non-reinforced specimen, shear failure occurred after the propagation of several diagonal tension cracks in the entire section of the specimen. In the case of Beam 2 and Beam 3, which were reinforced with Fe-SMA strips, bending-shear crack patterns were predicted. Moreover, compared to Beam 3 with non-activated Fe-SMA strips, larger vertical cracks caused by the bending load occurred in Beam 3 with activated Fe-SMA strips. These failure and crack patterns were relatively similar to those of the results reported by Czaderski et al. [12].

**Figure 17:**
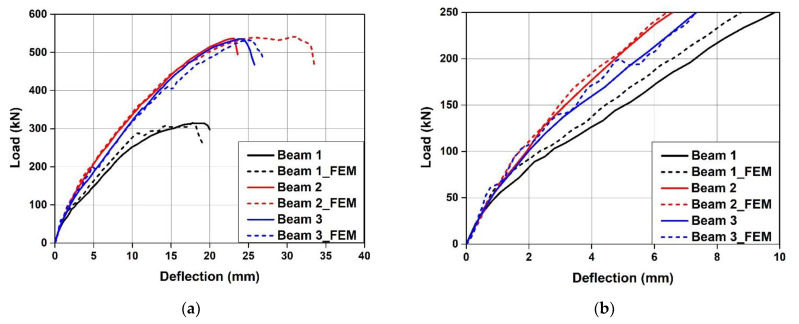
Comparison of load–deflection curves between FE model and experiment by Czaderski et al. [12]. (**a**) EXP and FEM. (**b**) Load-deflection curves at 200 kN.

Figure 17 compares the load–deflection relationships predicted by FE analysis with the experiment results reported by Czaderski et al. [12]. As can be seen from the figure, the load–deflection relationships of the specimens predicted by FE analysis were similar to those of the experiment results. In particular, the ultimate loads predicted through FE analysis differed from those of the experimental results only by 0.7% on average. This indicates that the proposed FE analysis model can predict the shear performance of the RC beam reinforced with Fe-SMA stirrups relatively accurately.

## 5. Conclusions

In this study, experimental and analytical research was conducted to evaluate the shear performance of a reinforced concrete (RC) beam constructed with Fe-based shape memory alloy (Fe-SMA) stirrups. The following conclusions were drawn.

1. Shear cracking in the RC beam was delayed by Fe-SMA stirrup reinforcement, and it was confirmed that the activation of Fe-SMA reduced the number of shear cracks. Therefore, it is judged that active shear reinforcement using Fe-SMA stirrups is effective in improving the usability of members through crack control.
2. Compared to the specimen reinforced with Fe-SMA stirrups at a spacing of 300 mm, the strain of the specimen reinforced at a spacing of 200 mm under a load increased by 52%. A decrease in reinforcement spacing from 300 to 200 mm increased the shear strength of the specimen by 27.1%.
3. The activation of the Fe-SMA stirrups increased the shear strength by approximately 7.6%. Therefore, it is judged that the introduction of active confining pressure through Fe-SMA stirrup activation is effective in improving the usability and shear strength of structural members.
4. The FE analysis model proposed in this study accurately predicted the failure mode of the RC beam actively confined through Fe-SMA, and the ultimate load of the members as predicted through the analysis differed from the experiment results by less than 5%. Therefore, the proposed FE analysis model can effectively predict the shear performance of RC members constructed with Fe-SMA stirrups.
5. The Fe-SMA stirrups used in this study can be constructed in the same manner as conventional shear reinforcement that uses rebar stirrups. The introduction of active confining pressure through Fe-SMA activation is expected to effectively improve the usability and strength of the structure.
6. In this study, a limited number of experimental variables such as the Fe-SMA stirrup activation and reinforcement spacing were considered for evaluating the shear performance of concrete beams reinforced with Fe-SMA stirrups. To address the limitations of this study, it is judged that future research that considers more variables such as concrete compressive strength, stirrup cross-sectional area, shear span ratio, heating temperature, and long-term effects should be additionally performed.

## Figures and Tables

**Table 1:** Test variables.

Specimen	Shear Reinforcement	Spacing (mm)	Activation
CTRL	-	-	-
N300	Fe-SMA stirrup	300	Non-Activation
A300			Activation
N200		200	Non-Activation
A200			Activation

**Table 2:** Material properties of the steel rebar used.

Standard	Nominal Diameter (mm)	Young’s Modulus (GPa)	Yield Strength (MPa)	Ultimate Strength (MPa)	Elongation (%)
D10	9.53	200	451	567	16.0
D13	12.7	200	462	540	17.1
D29	28.6	200	480	580	14.0

**Table 3:** Summary of test results.

Specimen	Cracking Load (kN)	Ultimate		Shear Strength		Deflection at 500 kN (mm)
		Load (kN)	Deflection (mm)	V_Total_ (kN)	V_Fe-SMA_ (kN)	
CTRL	144.16	437.08	3.8	218.54	-	-
N300	135.8	754	9.42	377	158.46	5.14 (100%)
A300	129.62	817.34	9.62	408.67	190.13	4.71 (91.6%)
N200	136.78	965.32	12.75	482.62	264.08	4.73 (92.0%)
A200	154.24	1031.28	12.91	515.64	297.1	4.41 (85.8%)

**Table 4:** Material properties used in the FEM.

**Concrete**						
Material model	MAT_SCHWER_MURRAY_CAP					
Density (kg/m^3^)	2400					
Compressive strength (MPa)	46.2					
Max. aggregate size (mm)	25					
Parameter	α	θ		β		γ
	13.373	0.33		0.025		6.89
	X	D1		D2		S
	104.12	6.11 × 10^−4^		2.225 × 10^−6^		2.0357
**Steel bars**						
Dimension	D10		D13		D29	
Material model	MAT_PIECEWISE_LINEAR_PLASTICITY					
Density (kg/m^3^)	7850					
Young’s modulus (MPa)	200,000					
Poisson’s ratio	0.3					
Yield strength (MPa)	451		462		480	
Tangent modulus	735		465		726	
Failure strain	0.16		0.171		0.14	
**Support and load rod**						
Material model	MAT_RIGID					
Density (kg/m^3^)	7850					
Young’s modulus (MPa)	200,000					
Poisson’s ratio	0.3					
**Fe-SMA stirrup**						
	Non-Activation			Activation		
Material model	MAT_ELASTIC_PLASTIC_THERMAL					
Density (kg/m^3^)	7850					
Young’s modulus (MPa)	98,684			111,642		
Poisson’s ratio	0.3					
Yield strength (MPa)	770					
Tangent modulus	1036					
Failure strain	0.17					
Coefficient of thermal	−21.4 × 10^−6^					

**Table 5:** Summary of FE analysis results.

Specimen	Ultimate Load			Deflection at Ultimate Load			Deflection at 500 kN		
	EXP (kN)	FEM (kN)	EXP/ FEM	EXP (mm)	FEM (mm)	EXP/ FEM	EXP (mm)	FEM (mm)	EXP/ FEM
CTRL	437.08	436.01	1.002	3.8	4.10	0.93	-	-	-
N300	754	788.63	0.96	9.42	9.23	1.02	5.14	4.49	1.14
A300	817.34	808.13	1.01	9.62	8.28	1.16	4.71	3.94	1.20
N200	965.32	992.49	0.97	12.75	15.58	0.82	4.73	3.97	1.19
A200	1031.28	1028.27	1.003	12.91	12.70	1.02	4.41	3.86	1.14

## Data Availability

Not applicable.
